# The Taverna workflow suite: designing and executing workflows of Web Services on the desktop, web or in the cloud

**DOI:** 10.1093/nar/gkt328

**Published:** 2013-05-02

**Authors:** Katherine Wolstencroft, Robert Haines, Donal Fellows, Alan Williams, David Withers, Stuart Owen, Stian Soiland-Reyes, Ian Dunlop, Aleksandra Nenadic, Paul Fisher, Jiten Bhagat, Khalid Belhajjame, Finn Bacall, Alex Hardisty, Abraham Nieva de la Hidalga, Maria P. Balcazar Vargas, Shoaib Sufi, Carole Goble

**Affiliations:** ^1^School of Computer Science, University of Manchester, Manchester, M13 9PL, UK, ^2^Astrazeneca, Alderly Park, Macclesfield, SK10 4TF, UK, ^3^Computer Science and Informatics, Cardiff University, Roath, Cardiff, CF24 3AA, UK and ^4^Faculty of Science, University of Amsterdam, 1098 XH Amsterdam, The Netherlands

## Abstract

The Taverna workflow tool suite (http://www.taverna.org.uk) is designed to combine distributed Web Services and/or local tools into complex analysis pipelines. These pipelines can be executed on local desktop machines or through larger infrastructure (such as supercomputers, Grids or cloud environments), using the Taverna Server. In bioinformatics, Taverna workflows are typically used in the areas of high-throughput omics analyses (for example, proteomics or transcriptomics), or for evidence gathering methods involving text mining or data mining. Through Taverna, scientists have access to several thousand different tools and resources that are freely available from a large range of life science institutions. Once constructed, the workflows are reusable, executable bioinformatics protocols that can be shared, reused and repurposed. A repository of public workflows is available at http://www.myexperiment.org. This article provides an update to the Taverna tool suite, highlighting new features and developments in the workbench and the Taverna Server.

## INTRODUCTION

The quantity and heterogeneity of data in the life sciences has given rise to thousands of Web Services that provide methods for its analysis, retrieval and integration (1). Large bioinformatics services providers, such as the EBI and the NCBI, routinely offer Web Service access to their resources in either REST or WSDL format (2). These Web Services can be executed and combined into multi-step analysis pipelines, or workflows, using systems like Taverna (3).

Workflows are reusable informatics analysis protocols. The myExperiment workflows repository (4) provides a collection of workflows [written with Taverna, or with other systems like Galaxy (5) or Kepler (6)] for use by the bioinformatics community. This collection of workflows provides a rich resource for scientists developing new analysis methods. Workflows can be combined and modified to assemble new executable protocols, using published and established pipelines as components.

In practice, most Taverna workflows are composed from a mixture of distributed Web Services, local scripts and other service types (e.g. BioMart queries or R-Scripts) (7). In some cases, Taverna workflows are only composed from local services, for example, where data and service execution must remain behind a firewall (e.g. clinical or commercial data), or where the size of data means that performance is significantly enhanced by reducing network traffic. For example, cloud installations of Taverna host the engine and services in the same cloud environment. Once an initial data set has been uploaded, the workflow engine and the services in the workflow can receive references to that data set, instead of the data itself.

The main advantage of using distributed services, however, is that the majority of computational processing in the workflow occurs remotely with the service providers. There is no requirement to install tools and data sources locally, which reduces local infrastructure and maintenance costs and enables rapid workflow development and testing. Consequently, genome-scale analyses can be performed regardless of local infrastructure, using distributed tools and resources.

A disadvantage of integrating third-party Web Services is the variable reliability of those services. If services are frequently unavailable, or if there are changes to service interfaces, workflows will not function correctly (8). There is, however, large redundancy in web service functions; therefore, the ability to identify reliable services and potential alternatives for non-functioning services is of great advantage. The BioCatalogue (http://www.biocatalogue.org) service registry provides this information, along with metadata descriptions of service inputs, outputs, dependencies and licenses.

This article is an update of a previous Web Services NAR special issue (3). Since this publication, the Taverna tool suite has undergone considerable changes and improvements, such as

- The implementation of a new Taverna engine (currently version 2.4) that caters for the scalable processing of large data sets, and it is capable of performing implicit iteration, looping and streaming of data.
- The ability to interact with new types of services in addition to WSDL Web Services, local scripts and BioMart data warehouses, in particular, RESTful Web Services, Grid Services, cloud services, R-scripts and distributed command-line scripts.
- The introduction of the myExperiment repository for sharing, reusing and repurposing workflows. Currently, myExperiment provides access to >2600 workflows.
- The introduction of the BioCatalogue and Biodiversity Catalogue service registries for the discovery and use of Web Services. They currently contain >2300 sets of Web Services, providing >8000 service operations.
- The introduction of the Taverna Server, which allows workflows to be executed on remote computational infrastructure (such as clusters, Grids and clouds), or as components in other workflow systems, such as Galaxy (9).
- The Taverna Player, an interface for the Taverna Server to allow workflow execution from web browsers, or through third-party clients.
- The Interaction Service, which enables scientists to select parameters and data during workflow execution.
- The Taverna Provenance suite, which records service invocations, intermediate and final workflow results and exports provenance in the Open Provenance Model format (http://openprovenance.org/) and the W3C PROV model (http://www.w3.org/2011/prov).
- Improvements to the plugin architecture to enable easier code contributions and extensions, making it possible to extend and personalize the core functionality to suit individual scientists.

## RUNNING TAVERNA WORKFLOWS WITH DISTRIBUTED SERVICES

Taverna Workflows can be designed and executed on local desktop machines through the Taverna workbench, or they can be executed through other clients or web interfaces using the Taverna Server (or the Taverna command-line application). These alternative execution modes serve different types of workflow users. The first execution mode is through the Taverna workbench. The workbench is downloaded to a local machine and provides an environment for bioinformaticians to develop new workflows and test new analysis methods, by either developing workflows from scratch, or by composing them from existing workflows.

The second mode of execution is simple execution through the Taverna Server. The Server is an environment for serving finished workflows to a larger community of scientists. In this mode, a single installation of the Server provides access to a collection of workflows (normally through a web interface, called the Taverna Player). Regular users are not required to download or install any software and do not require any detailed knowledge of distributed computing or Web Services. One drawback of this mode is that users cannot alter workflows or add new workflows to the collection. However, when workflows are being provided as a service, they require hosting on larger production-grade infrastructure to support multi-user executions, session management and potentially authenticated access.

The final execution mode is via a Taverna Lite installation. Taverna Lite provides an intermediate solution. It allows users not only to run workflows through the web but also to upload new workflows from myExperiment and other sources. Consequently, Taverna Lite installations require user authentication, but no local software installation by regular users, as workflow execution also occurs on a server. The following sections describe how to execute workflows using both the workbench and the Taverna Server.

### Using the Taverna workbench

The Taverna workbench is freely available and can be downloaded from http://www.taverna.org.uk/. It runs on Windows, Linux and Mac OS X. Installation is a one-click download, which must be unzipped on the local machine. For windows, there is also an installer wizard. There is no login required for the workbench, and the majority of third-party services in bioinformatics do not require a login. For those that do, Taverna allows credentials to be added at run-time, or to be stored in a purpose-built credential manager.

**Figure 1.**
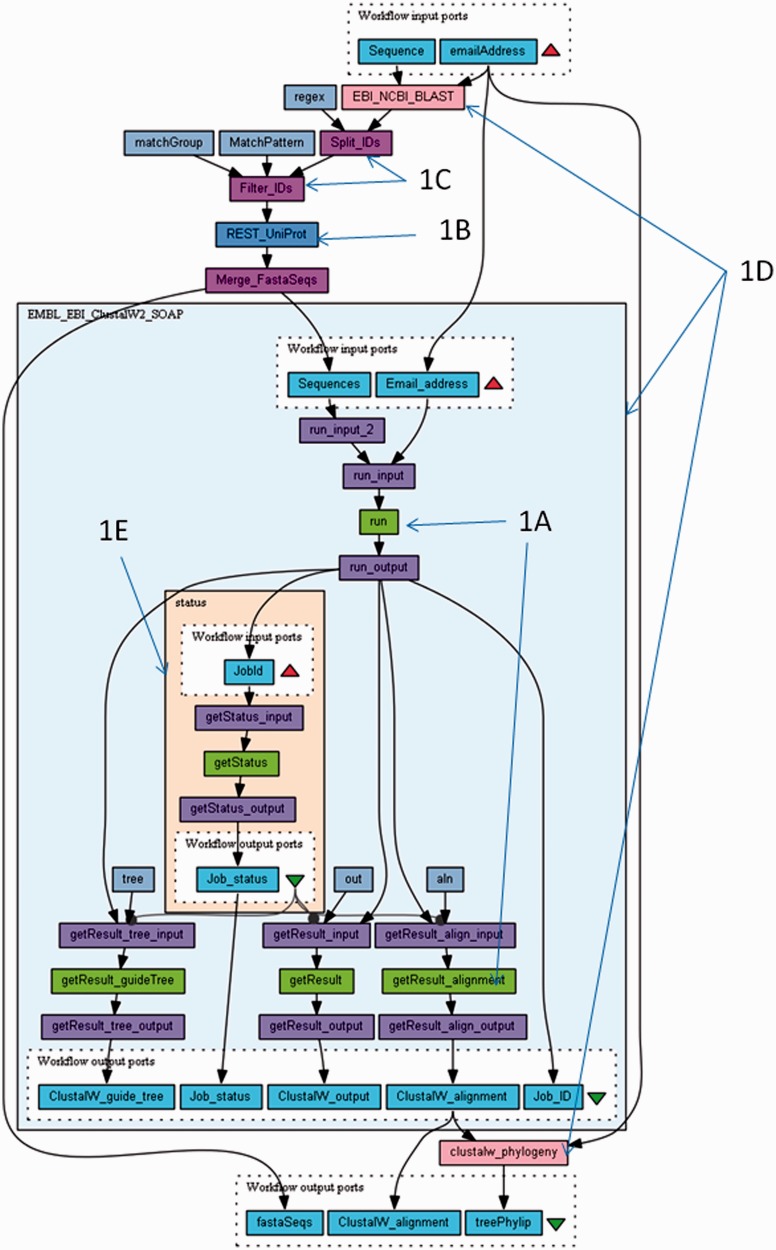
The Blast_Align_and_Tree workflow is an example Taverna workflow (available from http://www.myexperiment.org/workflows/3369.html), which performs a phylogenetic analysis. From an input protein sequence, it performs a similarity search against the Uniprot Database (10), using BLAST (11) and aligns similar sequences using ClustalW (12).The alignment is then used to construct a phylogenetic tree, using the EBI ClustalW phylogeny service. The workflow shows 1A = WSDL Web Services, 1B = REST Web Service, 1C = Shim service, 1D = nested workflows (both expanded and collapsed), 1E = asynchronous service looping.

The Taverna workbench allows users to identify and combine services by dragging and dropping them onto the workflow design panel. The Taverna quick start guide (http://www.taverna.org.uk/documentation/taverna-2-x/quick-start-guide/) provides step-by-step instructions on how to open and run existing workflows and how to design and run workflows from scratch. Figure 1 shows the Blast_Align_and_Tree workflow that features in the guide. It is part of the ‘Bioinformatics Workflow Examples’ pack (available on myExperiment at http://www.myexperiment.org/packs/363.html). This workflow performs a classic phylogenetics analysis. From an input protein sequence, it performs a similarity search [BLAST (11)] against the UniProt database (10) and aligns similar sequences using ClustalW (12). The alignment is then used to construct a phylogenetic tree using the EBI ClustalW phylogeny service (the workflow metadata describes the experimental methods in more detail).

This workflow predominantly uses Web Services from the EBI, in both WSDL (Figure 1A) and REST (Figure 1B) format, as well as local scripts for formatting data and managing service compatibility (also known as Shim services—Figure 1C). Almost all workflows require shim services because the analysis services have not been specifically designed to work together. Therefore, they often have incompatible input and output formats.

The workflow also contains several nested workflows (or subworkflows—Figure 1D), which can be downloaded individually from myExperiment, demonstrating that workflows can actually be components of other workflows. Some nested workflows control the retrieval of data from asynchronous services, using Taverna’s looping mechanism (Figure 1E). The nested workflow is executed repeatedly until results are available. Control links between the nested workflow output and downstream services pause the remainder of the workflow until all preceding results are available.

As the workflow runs, the results panel shows progress through the workflow and iterations over data. This view also displays any errors if there are problems with executions. The final workflow results show a list of similar protein sequences, the alignment of those sequences and a phylogenetic tree.

As of January 2013, myExperiment contains >1800 Taverna workflows, many of which are in the field of bioinformatics. The ‘Bioinformatics Workflow Examples’ pack is a collection of workflows that demonstrate specific features of the workbench, as well as a variety of bioinformatics analyses. Each workflow contains small sets of example input data, designed to produce results in a matter of minutes. Running these workflows on actual data sets may naturally take longer.

### Running workflows through the Taverna Server

The Taverna Server and web interface running at http://tavlite1.biovel.eu contain the workflows from the Bioinformatics Example Workflows Pack and additional workflows in the area of biodiversity. This server was developed by the BioVeL project (the Biodiversity Virtual e-Laboratory http://www.biovel.eu/). It provides informatics workflows for analysing biodiversity, using third-party Web Services in a widespread ‘service network’. There is no login required to use this server to execute a collection of public workflows, but only members of the BioVeL community have additional access to workflows under development and are able to submit new workflows to run on the server.

The BioVeL server instance is typical in that it provides a collection of workflows targeted to a particular research theme and provides a mixture of public and restricted access workflows. BioVeL biodiversity analyses include workflows for phylogenetics, metagenomics, population modelling and ecological niche modelling. For example, the matrix population model workflow enables the analysis of demographic data, such as age-specific survival, generation time or net reproductive rate. The workflow was developed from already published R-Scripts, using Taverna’s RServe service.

The functions of the workflows on the server can be explored through the ‘details’ links on the workflows page. The ‘run’ link allows workflow execution through the website. Each workflow is provided with default parameter values and example input data. The Blast_Align_and_Tree workflow has the same example data as in the myExperiment version and should, therefore, provide the same results thorough the web interface.

In general, the Taverna Server can be downloaded and configured to run with or without login restrictions. For a detailed description of installing the Server, see the beginner’s installation guide at: http://dev.mygrid.org.uk/wiki/display/taverna/A+Beginner%27s+Installation+Guide+to+Taverna+Server.

### The Taverna bioinformatics user community

There are Taverna workflows spanning most fields of bioinformatics, including omics analyses [such as transcriptomics, proteomics and metabolomics (13–16)], text mining, biodiversity and data integration (17,18). Taverna has also been adopted by a number of large-scale life science initiatives, such as the Virtual Physiological Human SHARE project, OpenTox (19) and caGrid (20). Most published workflows are available from myExperiment, where the number of views and downloads demonstrates the propagation of methods through the community.

Taverna is an open-source project. Community users have developed plugins to support specific user groups and tasks. For example, CDK-Taverna provides cheminformatics service support (21), and Tav4SB integrates tools for systems biology modelling (22). Other plugins support the use of Taverna on specific Grid or cloud resources, such as the UNICORE plugin (23), which enables users to submit jobs from the Taverna workbench to any UNICORE resource.

## DISCUSSION AND FUTURE WORK

Analysing and processing the wealth of available data is a central concern in the life sciences. However, accessing distributed resources, which are sometimes incompatible and liable to change over time, is challenging. Continuing and future work with Taverna focuses on managing the heterogeneity and ever-changing nature of distributed services. This includes researching workflow preservation and methods for aggregating collections of data, protocols and provenance into digital bundles, termed ‘research objects’. It also involves developing methods for producing compatible collections of services by wrapping Web Services and shim services into plug-and-play components. By supporting service discovery, workflow design, reuse and execution, the Taverna tool suite enables the exploitation of distributed bioinformatics data and analysis methods.
